# Six-week high-intensity exercise program for middle-aged patients with knee osteoarthritis: a randomized controlled trial [ISRCTN20244858]

**DOI:** 10.1186/1471-2474-6-27

**Published:** 2005-05-30

**Authors:** Carina A Thorstensson, Ewa M Roos, Ingemar F Petersson, Charlotte Ekdahl

**Affiliations:** 1 Spenshult Hospital for Rheumatic Diseases, Halmstad, Sweden; 2 Dept of Rheumatology, Lund University, Lund, Sweden; 3 Dept of Orthopedics, Lund University, Lund, Sweden; 4 Dept of Physical Therapy, Lund University, Lund, Sweden

## Abstract

**Background:**

Studies on exercise in knee osteoarthritis (OA) have focused on elderly subjects. Subjects in this study were middle-aged with symptomatic and definite radiographic knee osteoarthritis. The aim was to test the effects of a short-term, high-intensity exercise program on self-reported pain, function and quality of life.

**Methods:**

Patients aged 36–65, with OA grade III (Kellgren & Lawrence) were recruited. They had been referred for radiographic examination due to knee pain and had no history of major knee injury. They were randomized to a twice weekly supervised one hour exercise intervention for six weeks, or to a non-intervention control group. Exercise was performed at ≥ 60% of maximum heart rate (HR max). The primary outcome measure was the Knee injury and Osteoarthritis Outcome Score (KOOS). Follow-up occurred at 6 weeks and 6 months.

**Results:**

Sixty-one subjects (mean age 56 (SD 6), 51 % women, mean BMI 29.5 (SD 4.8)) were randomly assigned to intervention (n = 30) or control group (n = 31). No significant differences in the KOOS subscales assessing pain, other symptoms, or function in daily life or in sport and recreation were seen at any time point between exercisers and controls. In the exercise group, an improvement was seen at 6 weeks in the KOOS subscale quality of life compared to the control group (mean change 4.0 vs. -0.7, p = 0.05). The difference between groups was still persistent at 6 months (p = 0.02).

**Conclusion:**

A six-week high-intensive exercise program had no effect on pain or function in middle-aged patients with moderate to severe radiographic knee OA. Some effect was seen on quality of life in the exercise group compared to the control group.

## Background

Exercise is considered to be one of the most important treatments for patients with mild to moderate knee osteoarthritis [1,2]. Positive effects on pain and function, as well as cost-effectiveness have been reported [3,4]. The effect size obtained on pain experience is similar to that of pharmacological treatment [3,5]. The side effects have also been reported to be favorable, including reduced risk of inactivity-related disorders, such as cardiovascular disease and diabetes [2,6,7]. Moderate levels of physical activity are not associated with radiographic progression, but activities involving a risk of severe knee injury are closely related to increased risk of developing radiographic knee osteoarthritis [8-12]. The dose-response relationship of exercise on symptoms and function is not clear and exercise recommendations in osteoarthritis guidelines are based mostly on studies on elderly people, i.e. mean age ≥ 65 [3,13-15]. It is not clear whether exercise has a similar effect on pain and function in middle-aged patients compared with elderly patients. The aim of this study was to examine the effects of a short-term, high-intensity exercise program in middle-aged subjects (age 36–65) with definite radiographic knee osteoarthritis on self-reported pain, function, and quality of life.

## Methods

### Subjects

**Figure 1:**
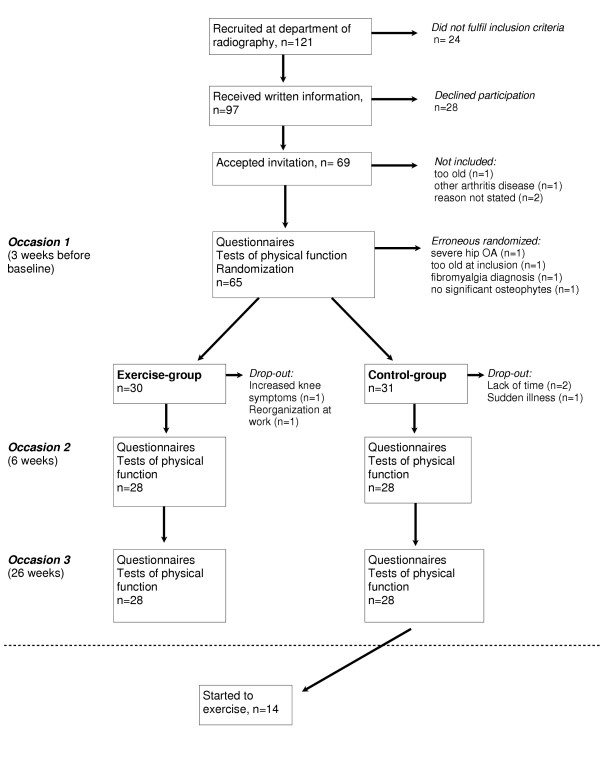
Flowchart of recruitment process and included patients.

A flow chart of the recruitment process is given in Figure 1. Radiologists and orthopedic surgeons at the Halmstad County Hospital, in the south-west of Sweden, and general practitioners within the catchments area of this hospital, were informed about the study and asked to list patients with radiographic knee osteoarthritis on a "patients eligible for research" list. Between October 1998 and October 2001 121 patients, referred by their general practitioner for radiographic examination because of long standing knee pain, were listed. Ninety-seven fulfilled the inclusion criteria: age 35–65, living in the defined geographic area, and diagnosis of radiographic osteoarthritis of Kellgren and Lawrence grade III or more, i.e. definite osteophytes and joint space narrowing. All listed patients received written information about the study. One week after the information was sent, patients were contacted by telephone, and invited to participate in the study. Twenty-eight patients declined participation for various reasons, the most common reason being lack of time and interest. To ensure only patients with symptoms due to knee osteoarthritis and eligible for exercise intervention were included, the following exclusion criteria were used: inflammatory joint disease, anterior cruciate ligament injury, known symptomatic injury to the menisci, hip symptoms more aggravating than the knee symptoms, about to have knee replacement surgery within 6 months, and co-morbidities not allowing exercise (Figure 1).

When eight or more patients fulfilled the inclusion criteria they were invited to baseline interview and examination for determination of exclusion criteria. Randomization was performed after the baseline examination. All patients were informed that they could be randomly allocated to either the exercise or the control group. After written informed consent, sixty-five subjects were randomized. Randomization was performed by the patient drawing a sealed envelope containing a piece of folded paper with either the word "exercise" or "control" written on it. Four persons were falsely randomized (one was too old at inclusion, one had severe hip osteoarthritis, one had fibromyalgia, and one had only joint space narrowing and no significant osteophytes), and thus 61 subjects entered the study. Thirty persons were allocated to the exercise group and 31 to the control group. Patients in the control group were offered exercise classes after the six-month follow-up period.

#### Exercise group

The number of participants exercising together varied from two to nine. There were eight intervention groups in all. One-hour exercise sessions, twice a week for six weeks, were supervised by a physical therapist (CT). The program consisted of weight-bearing exercises aimed at increasing postural control and endurance and strength in the lower extremity (see additional file 1 for the complete exercise program). Exercises were performed at five stations at submaximal intensity (minimum 60% of maximum heart rate (HRmax)). Intensity was gradually and individually increased during the six weeks by increased lever arms or range of motion. Patients were encouraged to exercise at their most vigorous intensity possible, without losing quality in performance or severely exacerbating pain. Pain during exercise was not considered as an obstacle if the patient perceived it as "acceptable" and no increased symptoms were persistent after 24 hours [16]. If pain exceeded this level, exercise intensity was reduced occasionally, until the "acceptable" level was found.

On every occasion, the heart rate of two random participants was estimated at each station using Polar pulsimeters (Polar^® ^Accurex Plus, Polar, Sweden). The other patients had their heart rate measured by the physical therapist or themselves, palpating their carotid arteries. Notes were taken by the supervising physical therapist, on every occasion and on all patients, about exercise intensity, heart rate, and perceived exertion according to Borg's Rate of Perceived Exertion scale (RPE) at each station [17]. These data were used to give the physical therapist a view of exercise intensity and to assure the preservation or increase of intensity from time to time. Patients were encouraged to keep up and increase intensity whenever possible throughout the six weeks.

Patients received a thera band to perform daily pulley exercises at home. In addition, three exercises, which were considered as the most challenging to the individual, were chosen as daily home exercises. Patients were recommended to perform some kind of weight bearing submaximal activity, such as walking or their home exercises, for at least 30 minutes or two times 15 minutes every day.

#### Control group

The controls were told not to make any lifestyle changes. They met the physical therapist (CT) for one hour at three times; baseline, follow up at 6 weeks and 6 months. After six months they were offered exercise classes or instructions and a home-exercise program.

### Outcome measures

#### Primary outcome

The primary outcome measure was the disease-specific Knee injury and Osteoarthritis Outcome Score (KOOS) [18,19]. The KOOS assesses the patients' self-report of pain, other symptoms, activities of daily living, sport and recreation function, and knee-related quality of life, in 42 questions which take about 10 minutes to complete. The KOOS is scored from 0 to 100, separately for each subscale, 0 indicating extreme problems and 100 indicating no problems. A change of 10 points or more is considered a clinically significant change [20]. The questionnaire and scoring manual can be found at . The Western Ontario and McMaster Osteoarthritis Index (WOMAC) [21] is included in the KOOS, and WOMAC scores can also be calculated.

#### Secondary outcome

Secondary outcome measures were the Short Form-36 item (SF-36), ergometer test, and five tests of functional performance. The SF-36 is a generic, widely used measure of general health status, which comprises eight subscales: Physical Functioning (PF), Role-Physical (RP), Bodily Pain (BP), General Health (GH), Vitality (VT), Social Functioning (SF), Role-Emotional (RE) and Mental Health (MH) [22]. The SF-36 is self-explanatory and takes about 10 minutes to complete. The SF-36 is scored from 0 to 100, 0 indicating extreme problems and 100 indicating no problems. The subscales assessing mainly physical components (PF, RP, BP, GH) were summarized to a physical component summary score (PCS), and the mental subscales (VT, SF, RE, MH) to a mental component summary score (MCS) [23]. Values are norm-based scored, using the U.S. general population norms from 1998. Each scale has the mean of 50 and standard deviation of 10. Scale score below 50 indicates a health status below average, and a score above 50 indicates a health status above average. Questionnaires were distributed prior to randomization at baseline, after 6 weeks, and 6 months.

**Figure 2:**
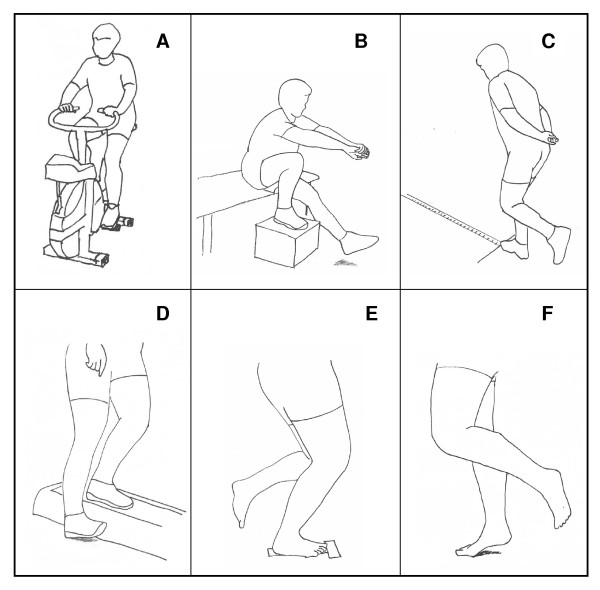
**Tests of functional performance**. A) Åstrand's cycle-ergometer test [24]. B) Rising on one leg from sitting on lowest possible height [25,26]. C) One-leg hop [25], [27]. D) Lateral step-up [28]. E) One-leg semi squatting; maximum number during 30 sec. [26]. F) Heel-rising on one leg; maximum number during 20 sec. [26], [29].

A bicycle ergometer test and five tests of functional performance were assessed (Figure 2).

1. Åstrand's bicycle-ergometer test [24] (Fig 2A).

2. Rising on one leg, from sitting on lowest possible height [25,26] (Fig 2B).

3. One-leg hop [25,27] (Fig 2C).

4. Lateral step-up [28] (Fig 2D).

5. One-leg semi squatting; maximum number during 30 seconds [26] (Fig 2E).

6. Heel-raising on one leg; maximum number during 20 seconds [26,29] (Fig 2F).

Tests of functional performance were recorded on three occasions: prior to randomization at baseline, after 6 weeks, and at 6 months.

To assess compliance the number of exercise occasions attended was noted.

### Statistics

Post-hoc, a power analysis was performed to estimate the number of patients needed to show a clinically significant difference between groups. Estimating the least clinical significant difference to be 11 ± 15 KOOS points, a total of 30 subjects in each group were needed to detect a difference with 80% power, p = 0.05.

Data were analyzed using nonparametric tests. P-values of less than or equal to 0.05 were considered to be significant, and all tests were two-tailed. To compare groups, Mann-Whitney U-test was used. Friedman's test was used for repeated measures analysis of variance. Six weeks was considered as the time-point of primary interest, and 6 months as follow-up. Wilcoxon signed rank test was performed to compare changes from baseline to six weeks and 6 months respectively. Analyses were performed using SPSS 12.0.1 for Windows [30].

The study was approved by the Research Ethics Committee at Lund University, Sweden (LU 99–98), and is in compliance with the Helsinki Declaration.

## Results

### Subjects

The mean age of the 61 included subjects was 56 ± 6 years, and the mean BMI was 29.5 ± 4.8 kg/m^2^. Patient characteristics are shown in table 1. Twenty-eight patients in each group were available for follow-up. The reasons for dropout were lack of time, reorganization at work, sudden illness, and increased knee symptoms (Figure 1). There were no clinically significant differences in baseline characteristics between the groups. Patient characteristics are shown in table 1.

**Table 1:** Patient characteristics at baseline

	Exercise group n = 30	Control group n = 31	p-value
Age (years) mean ± SD (range)	54.8 ± 7.1 (36–64)	57.3 ± 4.7 (46–65)	0.16
Gender number (%) women	15 (50%)	16 (52%)	0.90
BMI (kg/m)^2 ^mean ± SD	29.6 ± 4.5	29.5 ± 5.1	0.78
Aerobic capacity (ml O_2_/kg^x^min) mean ± SD	25.9 ± 6.4	25.2 ± 4.9	0.66
KOOS* pain	60 ± 18	64 ± 19	0.38
KOOS* symptoms	63 ± 20	66 ± 18	0.67
KOOS* ADL	69 ± 18	71 ± 21	0.76
KOOS* sport & recreation	34 ± 31	37 ± 29	0.54
KOOS* QOL	40 ± 15	46 ± 21	0.31

Knee Injury and Osteoarthritis Outcome Score [18,19]

Score from 0–100, worst to best.

### Compliance

The total number of performed supervised exercise sessions by the 28 patients available for follow-up in the intervention group was 302/336 (89.9%). Patients participated on average in 11 out of 12 possible exercise classes (12 classes (n = 11), 11 (n = 9), 10 (n = 6), 9 (n = 1), 2 (n = 1)). The most common reason for absence was illness not related to knee osteoarthritis, and work-related lack of time.

### Between-group differences

There was no difference between groups in pain or self-estimated function at either 6 week or 6 month follow-up. Quality of life improved significantly in the exercise group compared to the control group at 6 weeks (4.0 vs. -0.7, p = 0.05) and the results persisted at 6 months (5.1 vs. -2.3, p = 0.02, Table 2)

**Table 2:** Comparisons of change in Knee injury and Osteoarthritis Outcome Score (KOOS)^‡ ^[18,19] subscales between exercise and control group

KOOS Subscales		Exercise group		Control group		
		
		Change in KOOS score*	95 % CI	Change in KOOS score*	95 % CI	p^†^
Pain	6 w	1.8	-3.2 – 6.8	-0.3	-6.2 – 5.7	0.49
	6 m	3.1	-1.9 – 8.2	-1.1	-6.6 – 4.4	0.32
Symptom	6 w	0.2	-5.1 – 5.6	-3.8	-7.7 – 0.0	0.07
	6 m	1.0	-3.8 – 5.8	-3.4	-8.8 – 1.9	0.31
ADL	6 w	2.0	-2.3 – 6.3	-0.6	-7.0 – 5.8	0.96
	6 m	0.9	-3.8 – 5.6	-1.9	-7.7 – 3.9	0.61
SportRec	6 w	1.2	-7.9 – 10.4	-4.4	-12.6 – 3.7	0.22
	6 m	0.5	-10.1 – 11.2	-8.3	-19.5 – 2.8	0.32
QOL	6 w	4.0	-0.4 – 8.5	-0.7	-5.6 – 4.3	0.05
	6 m	5.1	-0.7 – 11.0	-2.3	-9.5 – 4.9	0.02

* negative = worsening, positive = improvement

^†^p-value for between group differences in change over time

^‡ ^[18,19]

**Figure 3:**
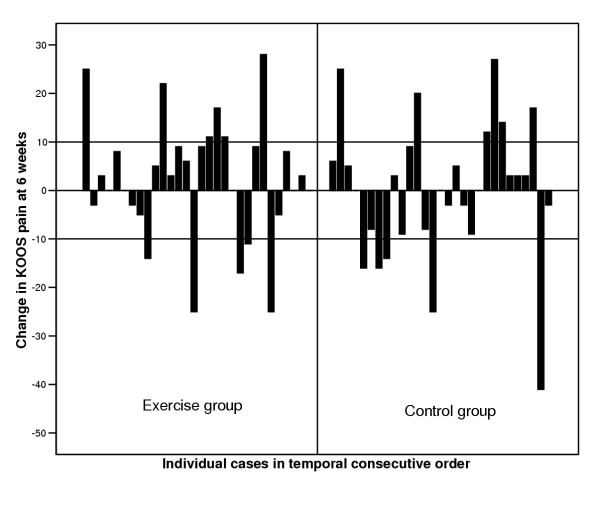
**Change in KOOS pain score at six weeks**. The individual change in KOOS pain at 6 weeks compared to baseline ranged from improvement to worsening, in both exercise and control group. A change of 10 points or more is considered clinically significant [20]. A similar pattern was seen for all KOOS and SF-36 subscales.

The individual differences ranged from clinically significant improvement of at least 10 points to clinically significant deterioration in all KOOS subscales and in both the exercise and control group (Figure 3).

#### Secondary outcomes

A significant improvement was found in the exercise group compared to the control group at six weeks with regard to the SF-36 Mental Component Summary scale (MCS) (2.1 vs -1.6, p = 0.04). At six months follow-up this difference was no longer persistent (Table 3).

**Table 3:** Comparisons of change in SF-36 Physical and Mental Components Summaries (PCS and MCS) [23] between exercise and control group.

Short Form-36 item (SF-36) [22]		Exercise group		Control group		
		
		mean	95%CI	mean	95%CI	p*
Physical Component Summary (PCS)	Baseline	42.5	24.4 – 57.5	43.8	24.2 – 57.3	0.49
	Change at 6 weeks	3.0	-5.9 – 13.4	0.3	-15.2 – 12.6	0.13
	Change at 6 months	3.0	-5.9 – 16.3	-0.7	-14.8 – 9.8	0.09

Mental Component Summary (MCS)	Baseline	55.6	40.2 – 66.2	56.3	37.0 – 67.0	0.63
	Change at 6 weeks	1.6	-10.6 – 15.0	-2.1	-16.9 – 11.5	0.04
	Change at 6 months	0.7	-18.1 – 13.2	-0.7	-16.8 – 12.8	0.40

* Comparison between groups

Improvements in functional performance of 0–20 % were seen in both groups at six weeks and six months. There was no difference in improvement between exercisers and controls (p = 0.08–0.9). See additional file 2 for the change in functional performance.

## Discussion

### Main message

Six weeks of intensive exercise had no effect on self-reported pain or function in middle-aged patients with symptomatic and moderate-severe radiographic knee osteoarthritis.

### Comparisons with other studies

Quite opposite to previously published studies on exercise in knee osteoarthritis we found no improvement in pain or function. Possible reasons for this include our study group having moderate to severe osteoarthritis compared with mild to moderate in most previous studies, being younger than previously studied groups and the intervention being of comparably high intensity.

It has been suggested that the responsiveness to exercise is modified by the loss of joint space width [31]. The homogeneity of this study population, with regard to radiographic changes, provided us the possibility to study the effects of exercise on patients with moderate to severe radiographic knee osteoarthritis. Can significant improvements of pain or self-reported function be expected in patients with radiographic knee osteoarthritis corresponding to Kellgren & Lawrence grade 3 or more? In this study, no improvements were seen on group level in pain or function. However, regular exercise in general is important to prevent diseases caused by inactivity [6], and thus patients with knee osteoarthritis should be encouraged to exercise. In clinical practice, patients with severe knee osteoarthritis should have treatments based on individual preferences and different stages of motivation [32].

It can be argued that the exercise intensity was too high for this group with moderate to severe knee osteoarthritis. Even though the intensity of each exercise was individually adapted, all individuals exercised at a minimum of 60% of HR max. It has been suggested that pain during exercise might be a protective mechanism in knee osteoarthritis, i.e. an increase in pain from too intensive exercises may restrain patients from further joint loading, which otherwise could cause further cartilage damage [33]. Patients in the current study were told to reduce the exercise intensity if pain during exercise was perceived as worse than 'acceptable', or persisted more than 24 hours.

It is suggested that the different degrees of varus-valgus laxity should be taken into account in exercise interventions, to enhance the functional outcome [34,35]. Severe knee osteoarthritis is associated with a hip-knee-ankle malalignment and an increase in varus-valgus laxity compared to healthy knees [36]. It is possible that varus-valgus laxity mediated the effect of exercise on pain since all patients had radiographic changes corresponding to Kellgren and Lawrence grade III or more. Malalignment may cause increased joint loads, and greater quadriceps strength might further increase joint load by the muscles compressing the articular surfaces [37].

Younger patients are usually more physically active than elderly [38], and have higher demands on level of physical function and physical performance at work or leisure time. Thus, moderate to severe knee osteoarthritis might be perceived as more disabling by younger individuals compared to elderly. Our study population was younger (<65 years) and comprised more men (49%) than most other populations with knee osteoarthritis described in randomized controlled trials of exercise [13-15,39,40], which might have reduced the effect on self-reported function in the present study.

This study showed no significant differences on self-reported pain and function either between or within groups. A post-hoc analysis was performed to study the possibility that the benefit from exercise was larger in subjects with worse pain at baseline. Fifteen patients in the exercise group were compared to 13 from the control group who had worse than total group median pain score (KOOS Pain 58 on a 0–100, worst to best scale) at baseline. The groups had comparable patient characteristics. The changes seen in these subgroups were however not different from the changes seen in the total groups.

A possible limitation could be lack of power. A post-hoc analysis was performed to estimate the number of patients needed to show a clinically significant difference of 11 ± 15 KOOS-points [20]. The standard deviation of 15 is supported by results from randomized controlled trials of glucosamine supplementation [41] and a nutritional supplement [42] for knee osteoarthritis, where significant group differences were found in KOOS pain and ADL subscales. The number of subjects in each treatment arm in these RCT:s ranged from 15 to 27. The standard deviations in KOOS subscales have not previously been determined in exercise interventions.

Only one of the five KOOS subscales showed a statistical significant improvement, and it can not be excluded that this result could be due to chance. The improvement of the KOOS subscale Quality Of Life in the exercise group was of doubtful clinical significance, however the improvement persisted over time, and is in accordance with previous findings of impact from exercise on mental health aspects in patients with knee osteoarthritis [31,43,44]. Group dynamics, support, or attention received may possibly have influenced the quality of life more than the exercise itself in the present study. Psychosocial factors are important determinants of physical function [45], and our results suggest that supervised exercises and follow-up are important, and that quality of life should be evaluated in osteoarthritis interventions.

## Conclusion

A six-week high-intensive exercise program had no effect on pain or function in middle-aged patients with moderate to severe radiographic knee OA. Some effect was seen on quality of life in the exercise group compared to the control group.

## Competing interests

The author(s) declare that they have no competing interests.

## Authors' contributions

CT participated in design, exercise intervention, assessments and follow-ups, statistical analyses and writing. ER participated in design, analysis and interpretation of the data, and critically revised the article. IP participated in design and interpretation of the data and critically revised the article. CE initiated and obtained necessary permissions for the study, arranged the initial funding, and participated in the analyses, interpretation and revision of the manuscript. All authors read and approved the final manuscript.

## Pre-publication history

The pre-publication history for this paper can be accessed here:



## Supplementary Material

Additional File 1 Intensive exercise program Carina Click here for file

Additional File 2 Tests of functional performance Carina Click here for file
