# Supplementary material for: Six-week high-intensity exercise program for middle-aged patients with knee osteoarthritis: a randomized controlled trial [ISRCTN20244858]
Source: BMC Musculoskelet Disord. 2005 May 30;6:27. doi: 10.1186/1471-2474-6-27 (PMC1187893; doi:10.1186/1471-2474-6-27)
Supplement: Additional File 1 — Intensive exercise program Carina [file 1471-2474-6-27-S1.pdf]

HIGH-INTENSITY EXERCISES FOR PATIENTS WITH KNEE OSTEOARTHRITIS

The program consisted of weight-bearing exercises for the lower extremities. They were performed in five stations at submaximal intensity.
Exercises aimed at increasing endurance and strength in proximal muscles and lower extremities.
Intensity: at least 60% of HRmax
Timewaste: 45 minutes + stretching.
Exercises were supervised and performed twice a week for six weeks. Furthermore patients were recommended to exercise at least 30 minutes a day in some kind of weight-bearing, submaximal activity, such as walking.

Pain during exercise was not considered an obstacle as long as it was below 5 on a Visual Analog Scale (VAS), or considered “acceptable” by the patient, and had disappeared within 24 hours after exercise. If pain was experienced as more than 5 on VAS, exercise intensity was reduced occasionally, until pain was experienced as “acceptable”.

The load of each exercise was chosen by the patient, but they were encouraged to exercise at the most vigorous intensity possible without losing quality in performance or severely exacerbating pain.

1. ERGOMETER CYCLING – 10 minutes

Intensity: 50–60 revs per minute,
load: self-chosen

2. TRAMPOLINE – 5 minutes

One of the following exercises, or a combination was chosen

- Tramping/walking
- Walk with high knee lifting
- Jogging
- Jumping – feet together or jogging with high knee lifting

3. STEP-BOARD – 5 minutes

One of the following alternatives was chosen and repeated 15 times per leg.

- Step up and down.
- Standing with one foot on the board. The other foot touched the floor with heel in front of the board and with toe behind the board alternately.

The height of the step-board was used to increase intensity.

4. FLOOR EXERCISES – 15 min

Each stage (A–D) was performed 15 times per leg at a time.

A) Hip lifting

Lying supine, one leg placed on a **square pillow**. The other leg flexed, with knee held against belly. Pelvis was lifted and lowered with maintained muscle control.

One of the following positions was chosen:

- calf-muscle rested on pillow
- foot placed on the center of pillow
- foot placed on the edge of pillow

B) Sit-ups

Lying supine, knees flexed and feet on the floor.

The most demanding exercise that could be performed with full muscle control was chosen:

- raising the shoulders, with hands reaching the knees/thighs
- raising the shoulders, with arms kept on the chest
- raising the shoulders, with hands held at the ears and elbows pointing laterally
- raising the shoulders, with arms held over the head along the bodyline

C) Hip abduction

Lying on side

One of the following exercises was chosen:

- Single straight leg rise (hip abduction). Hip extended and internal rotated.
- Standing on elbow and knee. Pelvis and upper leg were raised at the same time.
- Standing on hand and foot. Pelvis and upper leg were raised at the same time.

D) One-leg rise from sitting.

Sitting on a bench with adjustable height.

The lowest possible height from which both rising and descending could be performed with full muscle control was chosen. Weight was to be kept on foot during the entire movement. If one-leg rise was impossible, using both legs was allowed.

5. PULLEY – 10 minutes

Straight leg flexions, abductions, extensions and adductions of the hip, and lunging forward.

All five moments performed with 15 repetitions before changing leg.

Standing knee should be flexed about 10 degrees.

Loading was self-chosen. Maintaining performance quality throughout all stages was essential.

6. STRETCH – 10–15 minutes

- Mm triceps surae
- Mm quadriceps
- Mm hamstrings
- Mm iliopsoas
- ….

Patients were given a rubber-band to perform pulley exercises at home. Furthermore three of the most demanding exercises were chosen as daily individual home exercises. Patients were recommended to exercise at least 30 minutes, or two times 15 minutes, per day with these exercises and/or walking.
