# Supplementary material for: Six-week high-intensity exercise program for middle-aged patients with knee osteoarthritis: a randomized controlled trial [ISRCTN20244858]
Source: BMC Musculoskelet Disord. 2005 May 30;6:27. doi: 10.1186/1471-2474-6-27 (PMC1187893; doi:10.1186/1471-2474-6-27)
Supplement: Additional File 2 — Tests of functional performance Carina [file 1471-2474-6-27-S2.pdf]

**Change in functional performance in exercise and control group at 6 week and 6 month follow-up.**

| Test of physical function | | Exercise group | | | Control group | | | |
|---|---|---|---|---|---|---|---|---|
| | | n | mean | 95% CI | n | mean | 95% CI | p[†] |
| One-leg jump[*] cm | baseline | 29 | 54 | 9.2 – 125.8 | 28 | 38 | 3.4 – 98.1 | 0.04 |
| | Change at 6 w | 27 | 1.3 | - 27.7 – 35.8 | 25 | 3.1 | - 19.8 – 21.2 | 0.60 |
| | Change at 6 m | 28 | 2.3 | -34.0 – 51.1 | 26 | 1.3 | - 23.1 – 25.2 | 0.95 |
| Knee bendings/ 30 sec[*] | baseline | 29 | 22 | 10 – 34 | 28 | 21 | 8 – 40 | 0.38 |
| | Change at 6 w | 27 | 3 | - 9 – 10 | 24 | 2 | - 12 – 15 | 0.63 |
| | Change at 6 m | 28 | 4 | - 5 – 18 | 26 | 2 | - 11 – 15 | 0.08 |
| Toe raises/20 sec[*] | baseline | 29 | 17 | 9 - 27 | 28 | 15 | 6 - 29 | 0.34 |
| | Change at 6 w | 27 | 1 | - 6 – 8 | 24 | 3 | - 3 – 10 | 0.22 |
| | Change at 6 m | 27 | 1 | - 7 – 12 | 26 | 2 | - 6 – 13 | 0.46 |
| Lateral step-ups/30 sec[*] | baseline | 29 | 14 | 7 – 26 | 29 | 13 | 2 - 25 | 0.60 |
| | Change at 6 w | 27 | 2 | - 11 – 9 | 25 | 1 | - 5 – 8 | 0.13 |
| | Change at 6 m | 28 | 2 | - 9 – 7 | 26 | 1 | - 6 – 7 | 0.22 |
| One-leg rise[*] cm (lower value indicates better result) | baseline | 30 | 44 | 28 - 61 | 29 | 48 | 30 - 64 | 0.04 |
| | Change at 6 w | 27 | -1 | - 9 – 11 | 26 | 0 | - 9 – 11 | 0.13 |
| | Change at 6 m | 28 | 0 | - 9 – 8 | 27 | 1 | - 10 – 12 | 0.50 |
| Aerobic capacity $mlO_2/kg^x min$ | baseline | 27 | 25.9 | 16.2 – 41.8 | 24 | 25.2 | 11.5 – 33.2 | 0.66 |
| | Change at 6 w | 26 | 2.5 | -4.0 – 9.7 | 23 | 2.0 | -4.8 – 15.0 | 0.57 |
| | Change at 6 m | 27 | 2.1 | - 7.0 – 13.0 | 22 | 3.4 | - 9.1 – 18.5 | 0.40 |

[*]Mean of left and right leg
[†]Comparisons between groups
